# Patterns of acceptance and use of digital health services among the persistent frequent attenders of outpatient care: A qualitatively driven multimethod analysis

**DOI:** 10.1177/20552076231178422

**Published:** 2023-05-25

**Authors:** Lotta Virtanen, Anu-Marja Kaihlanen, Emma Kainiemi, Petra Saukkonen, Tarja Heponiemi

**Affiliations:** Welfare State Research and Reform Unit, 3837 Finnish Institute for Health and Welfare (THL), Helsinki, Finland

**Keywords:** Persistent frequent attenders, digital health, self-management, patient portals, electronic health records, telemedicine, patient experience, outpatient care, UTAUT

## Abstract

**Objective:**

Utilising digital health services in the treatment of patients who frequently attend outpatient care could be beneficial for patients’ health and the sustainability of health systems but carries the risk of digital exclusion. This study aimed to explore the patterns of acceptance and use of digital health services among frequent attenders (FAs), which may help in the assessment of patients’ digital suitability.

**Methods:**

Persistent FAs (*N* = 30) were recruited by random sampling from one Finnish municipality. The semistructured interviews were conducted in February–May 2021. We analysed the data with qualitative content analysis using the Unified Theory of Acceptance and Use of Technology (UTAUT) model. Additionally, we quantified the data for two-step cluster analyses to create separate cluster models that grouped FAs based on acceptance and use of (a) digital services for self-management of health and (b) telemedicine services.

**Results:**

Based on digital self-management, FAs were defined as *Self-Managers*, *Supported Self-Managers*, and *Non-Self-Managers*. Based on telemedicine use, they were grouped into *Telemedicine Users*, *Doubtful Telemedicine Users*, and *Telemedicine Refusers*. The clusters described different opportunities, awareness, and interest in using digital health services. Referral from professionals seemed to promote digital service use. For some, digital services were not accessible.

**Conclusions:**

Our findings emphasise the importance of assessing the suitability of FAs to digital health services, as their readiness to use may vary. Professionals should recommend digital services that support individual health to suitable patients. More accessible digital services could promote digital suitability despite functional limitations.

## Introduction

A small number of patients (1.6–3.6%) are known as persistent frequent attenders (FAs) who continue to visit outpatient care year after year and may account for 8–35% of all primary care visits.^1–3^ Several studies have attempted to describe FAs with the conclusion that they are a highly heterogeneous group.^1,2,4–10^ However, they have in common a chronic illness or multimorbidity, a mental health condition or disability, and the related continuous need for care due to acute exacerbations.^1,2,4–10^ Studies also suggest broader social and psychological needs and medically unexplained symptoms among FAs.^1,4,7,11^ With more complex care needs, continuity of care becomes increasingly important.^12^ The persistence of frequent attendance may indicate that FAs’ primary care is fragmented and not effective in meeting their needs and maintaining overall wellbeing. These reasons, in addition to long waiting times for primary care,^13^ can lead to seeking help elsewhere from the health system.^7^ For example, FAs are more likely to visit the emergency department for non-urgent needs than the general adult population.^14–17^ The current service behaviour of FAs imposes high costs on health systems and may divert scarce resources away from those whose needs could be met.^8,18^ The number of FAs in the population can be anticipated to increase as the population ages, and chronic conditions become more prevalent.^19^

The development of services for FAs to be more cost-effective and encourage self-care has been recognised as an urgent priority.^18,20,21^ One solution could be to promote the adoption of digital health services, including digital services for self-management of health and telemedicine services. These services could improve FAs’ opportunities to participate in planning and setting goals for care based on their needs, as well as receive timely care instructions.^22,23^ The resulting benefits could include preventing deterioration of health, reducing the need for physical visits at healthcare or demanding treatment, and maintaining overall wellbeing.^22,23^ Digital health services also aim to enhance communication between patients and professionals, which could promote continuity of care.^24^ Additionally, digital health services could compensate for inequalities in access to care, for example, due to waiting times, travel distance, or functional disability.^22,24^

Potential services for FAs’ self-management of health include mobile applications for tracking or managing personal health and patient portals and websites for self-care programmes, health information seeking, or health-related transactions such as renewing prescriptions and booking appointments.^25^ In general, improving patient activation has been associated with shared decision-making,^26^ improved health outcomes, and reduced service visits and costs.^27–29^ Evidence shows that the use of mobile health applications for chronic diseases has been associated with lifestyle improvements.^30^ There is still no strong evidence of the association between the use of patient portals and improved health outcomes or a decrease in service visits, but their use has been linked to improved quality of care as measured by patient satisfaction.^31^ Studies also suggest that people with chronic conditions or disabilities perceive digital services for self-management of health as more useful than the general population.^32–34^ Moreover, people with chronic conditions have perceived that real-time telemedicine services in interaction with healthcare professionals via phone, video call, or chat can facilitate access to care.^35–37^ In some studies, the use of telemedicine services in the treatment and follow-up of chronic conditions has been associated with health benefits.^35–39^

Despite the benefits, readiness to switch to digital health services may vary. Previous literature has suggested that technology users can be classified according to usage patterns into, for example, users, potential users, and non-users based on the needs, interests, or barriers they have to using the technology.^40^ The Unified Theory of Acceptance and Use of Technology (UTAUT) model specifies that acceptance and use of technology can be influenced by *facilitating conditions*, *performance expectancy* (that is, the additional value the patient expects to gain from the use), *social influence*, and *effort expectancy*.^41^ Additionally, *hedonic motivation*, in particular, has later been suggested as an essential construct.^42^ The UTAUT model has been successfully applied for studying the acceptance and use of digital health services among the general population^43,44^ and patients with specific chronic conditions,^45–47^ which has also shown that many patients lack the constructs that would promote use.

However, to our knowledge, the acceptance and use of digital health services by the unique patient group of FAs has not been studied before. Since previous studies on FAs in traditional health services have identified that their service needs are heterogeneous,^1,2,4–10^ it is necessary to expand the research to identify whether there are differences within the group regarding their potential to use different digital health services. Healthcare professionals’ possible false beliefs about the suitability of FAs for digital health services may guide possible incorrect decisions to refer to digital services. Referring unsuitable FAs to a digital service might undermine their adherence to care—for example, when digital skills are insufficient or the use of digital health services is not motivating—and increase service needs, costs, and the risk of digital exclusion.^48–51^ Likewise, excluding suitable FAs from digital health services could strengthen digital inequality and weaken the utilisation of the full potential of digitalisation in FAs’ care.

A qualitative research method would enable a patient-centred way to examine FAs’ acceptance and use of digital health services. Since the UTAUT model suggests that the adoption of digital services can be a complex phenomenon explained by several constructs,^41,42^ it would be useful to systematically structure the experiences of FAs. A multimethod approach would guide the systematic exploration of qualitative data by searching for patterns in the data using a statistical method.^52^ The resulting qualitative patterns that characterise and describe user groups could provide professionals and service designers valuable insights into patient-centred promotion of the use of digital health services among FAs. In other words, the grouping of FAs could help to understand for whom digital health services may be suitable and who may have limited opportunities to use them and how these limitations could be reduced.

This study aimed to explore the usage patterns for the acceptance and use of digital health services among the persistent FAs of outpatient healthcare. More specifically, we aimed to identify whether FAs can be divided into different subgroups by examining the presence of UTAUT constructs to explain the usage patterns for (a) digital services for self-management of health and (b) real-time telemedicine services.

## Methods

### Study design

This was an interview study that employed a qualitatively driven multimethod analysis. We quantified qualitative data to conduct cluster analyses, after which the results were interpreted qualitatively. Cluster analyses have been increasingly adopted from marketing to healthcare research to segment the patient population from quantitative data to inform practice about different health needs and target care strategies.^53,54^ The application of qualitative data to cluster analysis has been suggested to be robust even with small samples,^52^ but the use of such multimethod analysis has been so far scarce or limited to other disciplines.^52,55–57^ Our choice of cluster analysis as the method used was based on the aim of our study and the size and type of the data. Compared to many other dimensionality reduction methods, such as multiple correspondence analysis and factor analysis, cluster analysis was flexible in terms of sample size and allowed grouping based on the differential presence of all factors potentially contributing to complex phenomena,^52^ instead of modelling within-group variance using a smaller set of factors.

### Setting

We conducted the study in Finland, where the health system is primarily based on publicly funded health services to which patients have universal access.^58^ Public outpatient care services cover, for example, acute consultations, treatment of chronic diseases, some mental health services and health advice with a physician or nurse, screening and vaccinations, and outpatient specialist services by referral.^59^ Outpatient care is provided in municipal health centres, outpatient clinics, and hospitals or as home care services.^59^

Finland is a highly digitalised country,^60^ where the national healthcare guidelines recognise the benefits of digital health as an integrated care service, for example, in treating multimorbidity.^61^ National digital services that are potentially useful for FAs’ self-management of health are the patient portal (My Kanta Pages), digital self-care programmes (Health Village), digital health library (Terveyskirjasto), and digital symptom checkers (Omaolo).^62^ The provision of real-time telemedicine services is not nationally harmonised but depends on the service provider.^62^ The patient has the option of choosing whether to use the health service digitally. When the patient logs into a digital service for self-management of health, informed consent to accept the terms of use is asked based on legislation.^63^ Similarly, healthcare professionals have a legal obligation to request the patient's informed consent to provide telemedicine services.^64^

The use of digital health services has regularly increased in the population.^65^ Due to the COVID-19 pandemic, remote contacts between patients and primary care providers increased by eight percentage points from the beginning of 2019 to the end of 2020. In a recent population-level survey, approximately 50% of the adults had used digital services for self-management of health and 22% had used real-time telemedicine services.^65^ Internet use for other purposes was more common, as even 96% of the Finns aged 16–64 used the Internet daily, or almost daily.^66^ In the older age groups, 78% of those aged 65–74 were still regular Internet users, but their proportion decreased to 42% in those aged 75–89.^66^

### Recruitment of participants and data collection

We determined the persistent FAs as adults who had had at least eight annual visits in outpatient care, excluding emergency care, for at least 3 of the 4 years of follow-up (2017–2020), a definition based on previous studies.^3,67^ We obtained a random sample (*n* = 100) of FAs, including their names, mail addresses, and phone numbers, from registered data of one large municipality in Finland. Participation required informed consent and the ability to independently answer the interview questions on the phone, without, for example, the assistance of a relative. The sample size was guided by thematic saturation and the study aims.^68^

The first author (LV) conducted individual phone interviews with the participants between February and May 2021, during the second wave of the COVID-19 pandemic in Finland. The semistructured guide (see Supplemental Appendix A) included questions about using digital services for self-management of health and real-time telemedicine services during the past year. Additionally, experiences related to the use and perceived benefits of digital health services or reasons for not using or benefitting from them were asked. The guide allowed us to ask further questions about the relevant themes for a specific participant.^69^ The interviews were recorded into digital audio format with participants’ consent and professionally transcribed. The mean length of interviews was 29 min, and the transcribed text yielded a total of 299 pages of data (Times New Roman 12pt, 1.5 spacing).

### Data analysis

The data was analysed in a triangulated process.^52^ First, we conducted a qualitative content analysis by combining theory-driven and inductive analysis.^70,71^ The progress of the analysis is summarised in Supplemental Appendix B. The constructs of the UTAUT model (*use of digital health services*, *facilitating conditions*, *performance expectancy*, *social influence*, *effort expectancy*, and *hedonic motivation*) formed the main categories of the analysis. The transcripts were carefully read through, and the sets of ideas, in which participants had described one of the main categories according to the definitions of the UTAUT model,^41,42^ were extracted from them and summarised into reduced expressions (*n* = 224). Similar reduced expressions under the main categories were further grouped into subcategories (*n* = 12), whose final names were developed in the next phase of the analysis.

Second, the qualitative analysis was quantified by treating the subcategories as study variables and dividing the reduced expressions under each variable into yes or no type categories. We assigned a value of 1 if the participant had mentioned the presence of a theme related to the variable in the interview and 0 if the participant had mentioned its absence, or in some cases, if it had not been mentioned in the interview. For example, *digital support available* was coded as 1 = yes for participants who had support available and 0 = no for those who were not supported, whereas *digital health services promote the patient's active role* was coded as 1 = yes for participants who had mentioned the benefit or 0 = not mentioned. Age, gender, education, and health status were coded as background variables. Supplemental Appendix B provides a detailed description of the interpretation of qualitative data in the quantification process. At the end of the quantification, a validity check was performed to verify that the quantified profiles were compatible with the descriptions in the transcripts.

Third, we conducted two-step cluster analyses^72^ to distinguish patterns for the acceptance and use of (a) digital services for self-management of health and (b) real-time telemedicine services. Separate analyses were conducted: model A included *the use of digital services for self-management of health* and variables related to UTAUT constructs, and model B included *the use of real-time telemedicine services* and variables related to UTAUT constructs. The analysis considered the similarities and differences of all variables added in the models, but the relative importance of the variables in estimating the clusters differed. The resulting clusters were inspected by background variables. The two-step clustering algorithm first used a log-likelihood distance measure to distinguish a coarse set of subclusters based on the categorical data and then a probabilistic measure to group the subclusters into clusters.^72,73^ We assigned the analyses to a specific fixed number of clusters because we wanted to test different-sized cluster solutions to find the best model. The cluster solutions that received a ratio of sizes greater than three and the highest average silhouette coefficient (ASC) were considered valid.^74^ Values from 0.2 were preferred for ASC, implying the distinction in the distance among the clusters.^74^ Due to the qualitative nature of our study, we did not employ further statistical tests often preferred in large quantitative datasets.^72^ Atlas.ti version 9 and IBM SPSS Statistics version 28 were used for the analyses.

Fourth, we interpreted the clusters identified from the analyses qualitatively by returning to the qualitative categorisation and reduced expressions, which we also used to describe the clusters in the Results section.

### Ethical considerations

The study received ethical approval from the ethical board of the Finnish Institute for Health and Welfare (THL/4657/6.02.01/2020) and from the Finnish municipality that provided the register of FAs. The study obliges the ethical principles embodied in the Declaration of Helsinki.^75^ Each participant provided written or electronic informed consent by mail or email.

## Results

Table 1 shows descriptive statistics of the participants (*N* = 30) and study variables. The mean age was 65 (SD = 11.8), ranging from 23 to 84. Two-fifths had a complicated health condition, for which they needed assistance by living in sheltered housing, receiving home care, or attending rehabilitation. The majority (67%) had used digital services for self-management of health. These services included primarily digital patient portals, such as My Kanta Pages and municipal or occupational health portals, to check own health information, renew prescriptions, or schedule appointments. Other services used were Health Village for digital self-care programmes, digital health libraries such as Terveyskirjasto, and Omaolo digital symptom checkers, but their use was less common. None reported using mobile health applications. Only 37% had used real-time telemedicine services in which they interacted with a healthcare professional via phone or chat in a municipal or occupational healthcare digital service. No one had utilised video calls.

**Table 1.** Descriptive statistics of the participants (*N* = 30) and study variables.

Variable	Proportion, *n* (%)
**Background**	
Age group	
<60	7 (23.3)
60–69	11 (36.7)
≥70	12 (40.0)
Gender	
Men	17 (56.7)
Women	13 (43.3)
Education	
Low	16 (53.3)
High	14 (46.7)
Health status	
Complicated	13 (43.3)
Stable	17 (56.7)
**Use of digital health services**	
Using digital services for self-management of health	21 (70.0)
Using real-time telemedicine services	11 (36.7)
**Facilitating conditions**	
End devices available	24 (80.0)
Digital support available	13 (43.3)
Advanced digital skills	23 (76.7)
**Performance expectancy**	
Not needing digital health services	7 (23.3)
Digital health services save time and need for travelling	11 (36.7)
Digital health services promote the patient's active role	11 (36.7)
Digital health services support health	6 (20.0)
**Effort expectancy**	
Using digital health services requires high effort	14 (46.7)
**Social influence**	
Initiative to use digital health services came from others	8 (26.7)
**Hedonic motivation**	
Positive attitude towards digital health services	17 (56.7)

*Note.* The study variables described whether participants mentioned an existence (‘yes’) of a particular topic in the interviews. Their frequency distributions were calculated as numbers and percentages.

### Clusters for the use of digital services for self-management of health

**Figure 1.**
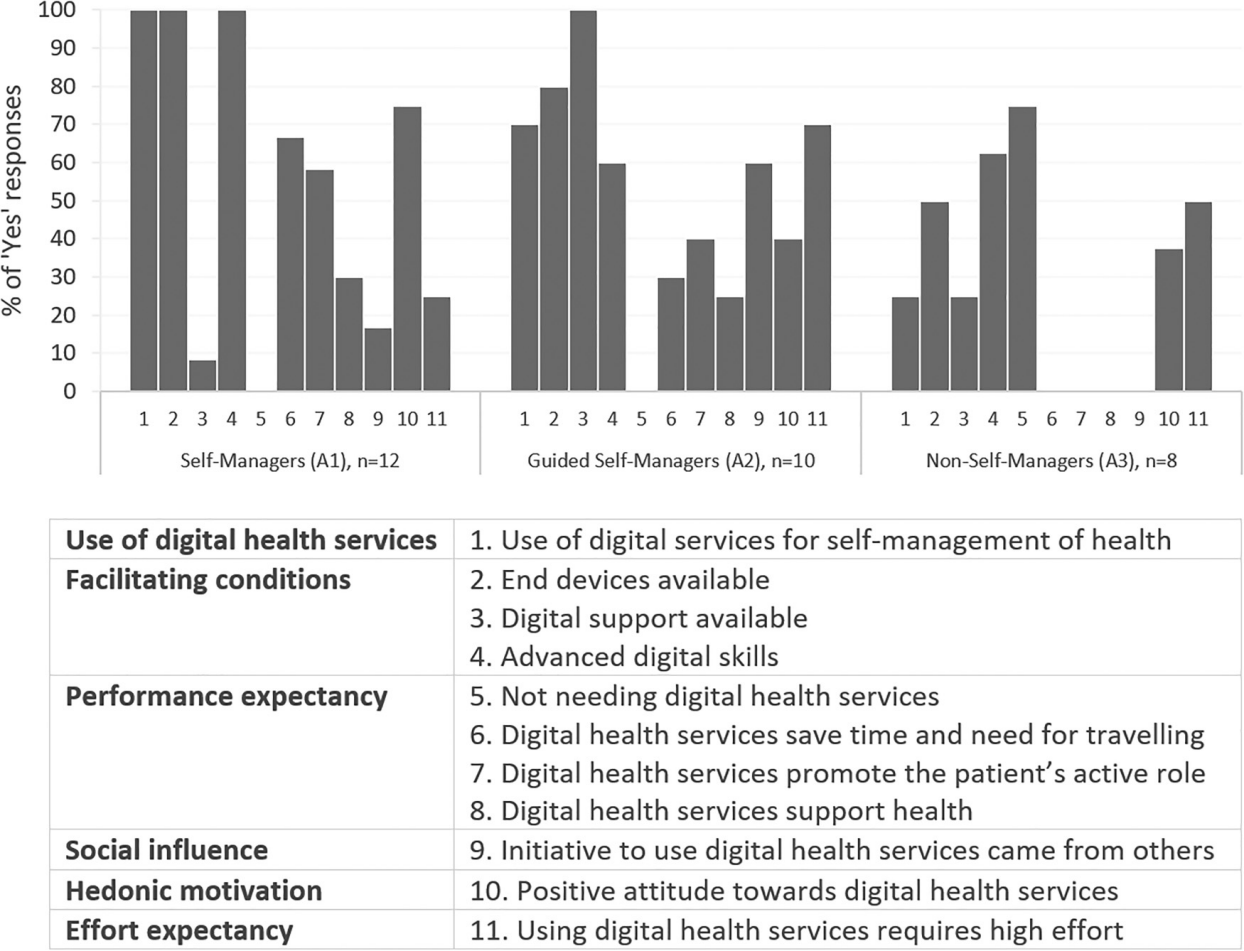
Possible clusters among FAs to characterise opportunities to accept and use digital services for self-management of health with a silhouette coefficient = 0.3 (fair). The figure shows the proportion of ‘yes’ responses for variables related to the UTAUT model (labelled as numbers 1–11) by cluster. FAs: frequent attenders; UTAUT: Unified Theory of Acceptance and Use of Technology.

As shown in Figure 1, our analyses resulted in three clusters for the use of digital services for self-management of health: (A1) *Self-Managers*, (A2) *Supported Self-Managers*, and (A3) *Non-Self-Managers.* The three most important variables for distinguishing the clusters (predictor importance ≥ 0.3) were as follows: *use of digital services for self-management of health*, *digital support available*, and *initiative to use digital health services came from others*.

#### Cluster A1: *Self-Managers*

*Self-Managers* (*n* = 12) had a relatively higher proportion of FAs aged at least 70 (50%) than other clusters. There were an equal number of FAs of both gender and levels of education, and 58% were in stable health. They all used digital services for self-management of health. These FAs were characterised by their motivation to self-manage their health digitally, which emerged from their expectations of gaining additional value. For example, many were using multiple medications and described that the use of patient portals had saved time as they could check the validity of their prescription and ask for a renewal. Thus, unnecessary travelling to the pharmacy or healthcare provider was prevented. Some also perceived that digital self-management services promoted their active role as a patient. They described that they had received transparent information concerning their visits to the healthcare provider at a rapid pace: ‘*Almost don’t even have time to get home when already can peek there and notice that everything that has been done has been recorded’* (A1#7).

Although some pointed out that the Internet might also contain false health information, they did not consider it a problem to search for trustworthy sources. These users described that searching and applying the newest health information from digital services had made them ‘almost like a physician’ themselves, which had promoted their health.

I can obtain up-to-date information from the digital health information service. I have these hereditary diseases myself, and the medicine and things like that are developing in the field all the time, so I’m regularly visiting there to check whether anything new has come up. (A1#5)

#### Cluster A2: *Supported Self-Managers*

*Supported Self-Managers* (*n* = 10) had a relatively higher proportion of FAs aged 60–69 (50%), who were men (70%), and in stable health (80%) compared to other clusters. They had equally low and highly educated FAs. Most of them already used digital services for self-management of health but found the independent use challenging. Challenges that had arisen included logging into digital services through electronic identification and navigating or completing tasks in these services. Some mentioned strong confidence in the security of digital services but were concerned about their poor data security skills that could jeopardise their sensitive health information. However, typical for these FAs was that digital support was readily available for them, provided most often by their relative, such as a spouse or child, but a few also mentioned a home care professional. They appreciated the provided support, as they also tended to perceive that using digital services for self-management of health required high effort.

I read all of my medical case summaries and check my laboratory results. So I’m a digital customer there and know how to type in there. But the reason behind it is that my spouse is so skilled in these computer things. So I’ve this good situation that I’ve them to help me. Otherwise, I’d experience a tough situation. (A2#8)

FAs from this cluster were also characterised by the strong social influence that encouraged them to use the services digitally. Most often, the initiative came from a healthcare professional, referring them, for example, to check the results of health examinations on the digital portal. Some described that their spouse was digitally active, which had encouraged them to try the digital services. Overall, with the available support, these FAs seemed to be relatively motivated to learn more about using digital services for self-management.

#### Cluster A3: *Non-Self-Managers*

*Non-Self-Managers* defined FAs (*n* = 8), of whom 38% belonged to the oldest age group, and 50% were women. Among them, there were relatively more low educated (63%) and of those with complicated health (50%), such as mild memory impairment and impaired vision, compared to other clusters. These FAs had relatively less experience with digital services for self-management of health compared to the other clusters, and the conditions that facilitate the use were also less prevalent. For example, they tended not to have any other end devices than smartphones, which they perceived challenging to use for services because the screen was too small to read the text or the functionality was poor on the phone.

These FAs were characterised by their tendency to expect no additional value from digital services for self-management of health. As a result, many mentioned that they perceived no need to use the services. For example, they stated that they saw no point in checking the results of health examinations or medical patient descriptions from the digital patient portal, as they received the same information by a post letter within a few weeks.

Often, or always, there will be information sent by letter about those control visits as well. So, why would I read the same things from the digital portal? I’ve created such a need-based basis for using the Internet so that I’ll learn and use only what I need. (A3#6)

Their knowledge of digital services also seemed to be limited to the patient portals, if at all. For example, those needing self-measurements in managing diabetes mentioned that they recorded the results in a paper booklet instead of mobile health applications. No social influence in encouraging the use was mentioned either.

Some in this cluster described a high threshold and low motivation to use or learn to use the services. However, a few members of the cluster anticipated that if digital support were available, they could be eager to learn.

I could be willing to learn if somebody would teach me. Suppose the teacher is a good one. In this sheltered housing where I’m living, there are nurses, but they don’t know how to teach or are not really willing either to teach me properly. (A3#4)

### Clusters for the use of real-time telemedicine services

**Figure 2.**
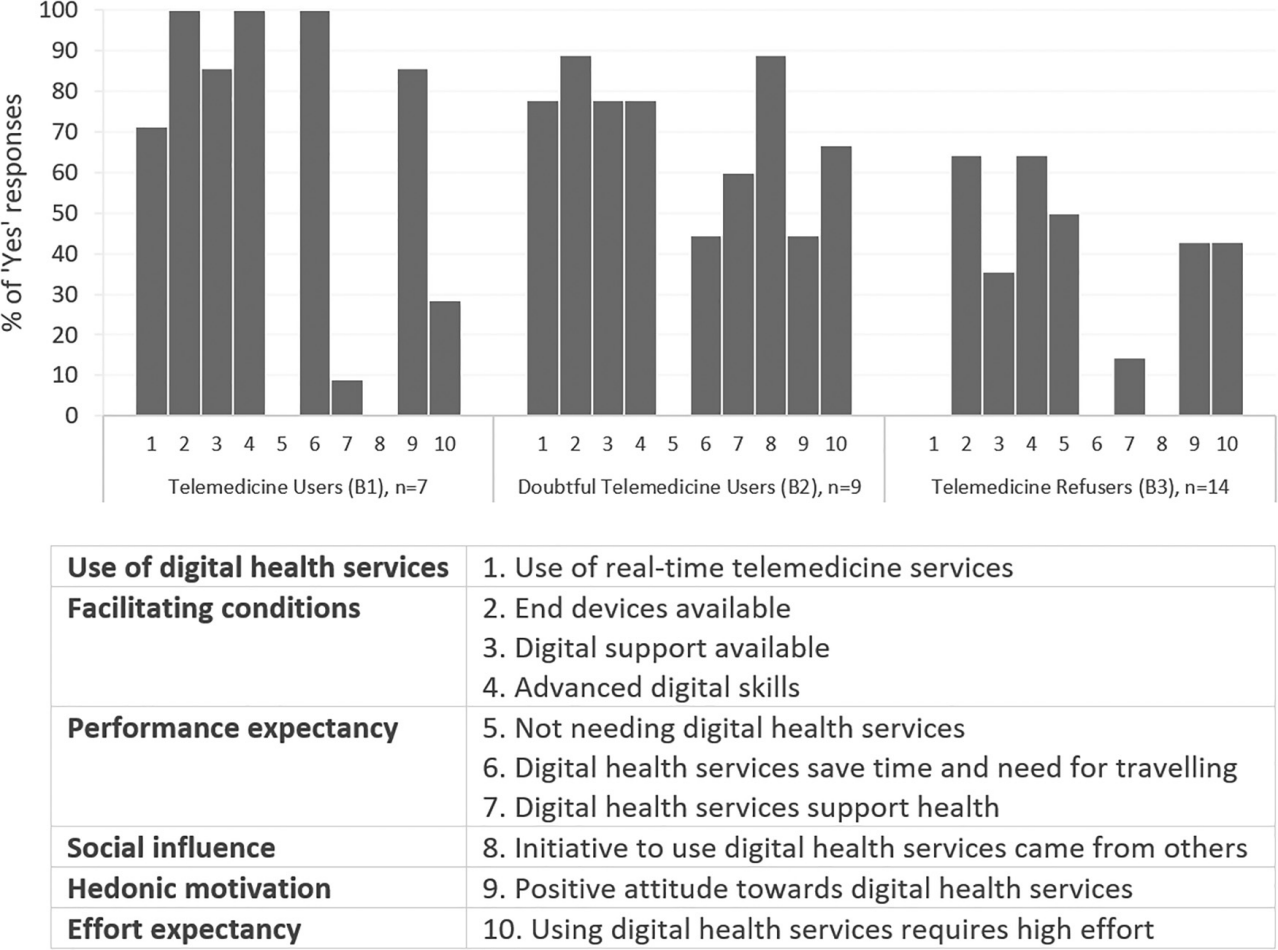
Possible clusters among FAs to characterise opportunities to accept and use real-time telemedicine services with a silhouette coefficient = 0.3 (fair). The figure shows the proportion of ‘yes’ responses for variables related to the UTAUT model (labelled as numbers 1–11) by cluster. The clusters were modelled without ‘digital health services promote the patient's active role’ because all the mentions in interviews regarding that variable were related to self-management and not to telemedicine. FAs: frequent attenders; UTAUT: Unified Theory of Acceptance and Use of Technology.

Figure 2 shows the three clusters identified to describe the use of real-time telemedicine services: (B1) *Telemedicine Users*, (B2) *Doubtful Telemedicine Users*, and (B3) *Telemedicine Refusers.* The three most important variables for distinguishing the clusters (predictor importance ≥ 0.6) were as follows: *digital health services save time and need for travelling*, *positive attitude towards digital health services*, and *advanced digital skills*.

#### Cluster B1: *Telemedicine Users*

*Telemedicine Users* included FAs (*n* = 7) who tended to be older (71% at least 70 years old), be women (71%), and have a lower educational background (71%) compared to other clusters. Most (71%) did not report having complicated health conditions; instead, they described being active in hobbies or at work. The use of real-time telemedicine over the phone and chat was familiar to these FAs. Although they had available end devices, including a web camera, they had only used video calls for leisure activities and socialising. Nevertheless, they seemed open-minded to trying a video connection to health services, too, as the comment below illustrates.

Yes, of course, I could use it. Because it would be easier, so if I’ve some rash or an injury like this and if I can show it through video, then the person at the other end of the connection can comment on what to do. Or whether there's a need to come to the healthcare centre. (B1#2)

These FAs were characterised by their perception that using real-time telemedicine services can save time and effort in travelling to a healthcare provider. One FA was even picturing this issue from the perspective of professionals.

Of course, it's nice when I don’t need to leave. I’m eager for flexibility. But I always think about the other side too. I don’t want to use the time of a physician or nurse in vain if it's a handy matter that can be handled on the phone. (B1#7)

They had experienced that using real-time telemedicine services was relatively easy for them. This became apparent, for example, from their perception that consulting a healthcare professional via telemedicine was comparable to those in-person visits not requiring physical examinations. Positive views seemed to boost motivation for the future use of real-time telemedicine services, although the FAs still wanted to prioritise in-person visits, especially in complex health issues.

#### Cluster B2: *Doubtful Telemedicine Users*

*Doubtful Telemedicine Users* consisted of FAs (*n* = 9) evenly from different age groups, of whom most were men (67%), had high education (67%), and stable health condition (78%). Most had used real-time telemedicine services and had the facilitating conditions for the use. However, these FAs stood out in their experience that discussing with a physician or nurse on the phone or chat had required much higher efforts than in-person health services. They perceived that it was more of their responsibility to remember to ask about or describe their health in telemedicine services. Some also shared that they had difficulty in self-expression in general. This disadvantage was only highlighted in telemedicine, as one user described: ‘*When it comes to describing things, it's kind of different remotely. As I’m pretty stiff, too. Bringing things out is not an easy task for me’* (B2#5).

Another user said there was always a familiar healthcare professional in in-person services, which was not the case for real-time telemedicine services. The need to repeat one's medical history to a new professional felt frustrating and even challenging, for example, due to memory impairment. Thus, real-time telemedicine services were found to be less ‘personal’ with lower possibilities of a holistic health assessment.

Of course, they don’t bother to read long care histories from my records. And neither am I bothering to tell everything every time if the professional changes there. I received those retirement papers due to memory problems, so yes, that's an issue too if I don’t remember; I remember what has happened roughly, but I cannot put it in chronological order. (B2#9)

The challenges concerning the efforts these FAs had to put into interaction appeared to undermine their motivation to use telemedicine. As a result, the desire to use the services in person recurred in the cluster, with a particular emphasis on face-to-face contact forming a crucial part of the care relationship.

#### Cluster B3: *Telemedicine Refusers*

The majority (*n* = 14) of the FAs belonged to the cluster of* Telemedicine Refusers*, of whom 50% were aged 60–69, and most were men (64%) and had low education (57%). FAs in this cluster had relatively more of those with complicated health (50%), such as mild memory impairment or speech production difficulty, than others. None of them had yet used real-time telemedicine services, and conditions facilitating the initiation of using them were also less present than those among FAs belonging to other clusters. For example, some pointed out that they did not have the end devices they thought were needed to use telemedicine services via video or chat. One FA described that with their small pension, it would also be utterly unfeasible to try to purchase all the ‘widgets’.

If I only had a better pension, I could buy a new computer where all these widgets could be installed so that it would be decent. But the computer I currently have, I’ve had it already 5 years and got it as a second hand; it has seen its days. The services would not work out. But try to go and buy a new computer with your 800 euros pension. (B3#12)

These FAs tended to have strong preferences against digital technology for real-time health services. However, some were more flexible with using the phone to obtain services. They explained their lack of motivation through their ‘old-fashioned mind’, which was brought up irrespective of their actual age. Some even argued that they would rather use only in-person health services ‘until the end’.

I prefer the old practice, to utilise an envelope or maybe a phone somewhat, but nothing digital for health services. It's best to go there in person. Maybe I could learn something new if I wanted to, but no, I am going with the in-person services that I have now, and I’m doing well. (B3#1)

Some mentioned that they lived so close to the healthcare provider or had received in-person services ‘whenever needed’ that they saw no point or need to use telemedicine services. Some also believed their service needs were too complex to reap practical benefits from real-time telemedicine services. Additionally, these FAs seemed unaware of the possibility of real-time telemedicine services because they had not been offered them as an alternative.

## Discussion

The FAs were grouped by their acceptance and use of services for digital self-management of health into *Self-Managers*, *Supported Self-Managers*, and *Non-Self-Managers.* Based on their acceptance and use of telemedicine services, we found *Telemedicine Users*, *Doubtful Telemedicine Users*, and *Telemedicine Refusers.* The identified clusters corroborate studies showing that FAs differ by sociodemographics and health and service needs,^1,4–9,18^ and add to those that FAs’ digital health behaviour also varies to a large extent. Thus, our study highlights the individual needs among this patient group, which should be considered in case management decisions.

We found that different conditions facilitated FAs’ potential for digital health services. For example, the availability of digital support distinguished the clusters in need of support, when *Supported Self-Managers* were able to use digital services to manage their health mainly with the help of their relatives, whereas no help was available for *Non-Self-Managers*. The importance of relatives in supporting use has also been previously recognised.^76–78^ However, informal support may introduce privacy threats, as support givers may see sensitive health information or identification to the service may require sharing bank IDs. In our study, FAs did not mention the threats—not even *Supported Self-Managers*, although previous studies have found it as an obstacle to asking for support from relatives.^78^ Not everyone may recognise the risks. Producers of digital health services should bear responsibility for providing secure digital support.

In our study, *Non-Self-Managers* and *Telemedicine Refusers* seemed to have relatively more FAs who did not experience that digital health services would add value to them and fewer of those with previous experience of using them. In turn, *Self-Managers* and *Telemedicine Users* stood out because they relatively often mentioned the benefits of digital health services, which were in line with those identified among chronically ill patients^45–47,79^ and in digitalisation goals.^22^ The differences in our clusters support previous research, according to which the perception of added value could be the primary determinant in adopting digital health services among the chronically ill.^45–47^

In our study, the presence or absence of social influence also appeared to be important in explaining the use of digital health services for those FAs who were not self-conscious about the services. For example, most *Supported Self-Managers* and *Doubtful Telemedicine Users* had already used digital health services, for which they said they had received an initiative from a healthcare professional or relative. In contrast, *Non-Self-Managers* and *Telemedicine Refusers* who used little or no digital health services lacked external initiative. This finding is congruent with previous studies where the recommendation by a healthcare professional or a trusted relative was associated with higher use of digital health services.^34,47,78,80^

Informing about the benefits of digital health services and encouraging their use is important for everyone, but our study suggests that FAs may have different information needs. Based on our results, digitally active *Self-Managers* could benefit from better information about digital services for self-management of health, as they mainly used services intended for the entire population instead of services that would support self-management of a specific illness. In turn, other groups had limited awareness of the benefits and the entire digital health service field. Healthcare professionals have a central position in responding to information needs so that services relevant to the patient's condition can be better utilised in promoting health. Previously, chronically ill patients have also expressed their willingness to be informed more about digital health services suitable for individual needs.^81^ In informing and engaging patients in digital health services, it is essential to earn their trust, understand individual situations, and strive to influence emotional awareness of illness and ability to maintain health.^18,82^ After being informed, the patient's right to decide how to use the service must be respected. Sufficient resources should be directed to the training of healthcare professionals to meet these competence demands.

*Self-Managers* and *Telemedicine Users* seemed to experience less challenges in the use of digital services and were thus also the most motivated clusters compared to others in our study. In other clusters, digital self-management seemed to be hindered by perceived poor accessibility or usability of services. The digital self-management of health for patients with, for example, mild memory impairment and impaired vision could be promoted by improving the readability of content and embedding slow-paced audio assistance, larger labels that can be pressed outside the visual layout, linear navigation, and centrally placed essential functions.^83,84^ Moreover, collecting feedback on the user interface could help develop more accessible services.^85^ The challenges expressed in telemedicine services were primarily related to the difficulties in expressing oneself and one's state of health. Some FAs with the onset of memory disorder experienced difficulties in describing their medical history in telemedicine services because they encountered unfamiliar professionals in them more often than in in-person services. Fragmented care, including often changing professionals, can be typical in the care of FAs.^7^ When used effectively, electronic health records should ensure the continuity of treatment-related information and management even without a long-term patient relationship.^86^ It would be essential to secure the continuity of care for FAs, as it may be associated with better care outcomes.^87^

Another explanation for the difficulties in expressing oneself may be related to insufficient telemedicine competence of professionals, such as a lack of remote interaction skills, as previously suggested.^88^ None of the FAs we interviewed had experience with video calls, although they could enhance the interaction by showing facial expressions and visible clinical symptoms. Video-based telemedicine has been studied with positive results in patients with mild cognitive symptoms, mild dementia, and aphasia.^89–91^ Therefore, video calls could facilitate the use of telemedicine services even among *Telemedicine Refusers* who struggled the most with memory or speech production. Instead of the patient's characteristics, the abilities and preferences of healthcare professionals may have had a stronger weight in the decision whether to provide a service by phone, video call, or in-person service,^92^ which should be paid attention to in healthcare organisations.

Our findings suggest that FAs are more open to digital services for self-management of health than telemedicine services. This is congruent with previous studies that found that most patients generally perceive that remote interaction with a provider cannot substitute for in-person health services.^47,79,93^ Interestingly, our findings suggest that this attitude may have persisted among FAs despite the COVID-19 pandemic when telemedicine could have benefitted many of them.^94,95^ Telemedicine in the routine care of FAs would still need development work to be comparable to in-person services. Integrating telemedicine peripherals,^96^ increasing the provision of video connection, and strengthening professionals’ telemedicine competence could be a place to start. For example, professionals should be supported in working in a ‘webside manner’, that is, communicating and using the tone of voice with patients in a way that makes the atmosphere comfortable and reliable,^97^ encouraging patients to speak up, and asking guiding questions.^98^

### Limitations and trustworthiness

There are some limitations to this study. The interview guide was not linked to the UTAUT model; thus, we relied on the participants’ expressions and our interpretation of their connection to the UTAUT constructs. However, this decision enabled participants to raise the topics they perceived most important and prevented leading phrasing of questions. The credibility of the results could have been enhanced if we had asked interviewees to check the accuracy of their own cluster profile.

The analysis was performed by one researcher, which is important to note as it may have biased results. However, to mitigate this, we held frequent discussions within the research team before and during the data analysis and strictly followed the coding scheme. The analysis process was also mainly based on seeking whether the construct based on the definitions in the UTAUT model was present for the participant or not, instead of interpreting more ambiguous topics. To increase trustworthiness, we aimed to establish a clear and logical connection between the original qualitative data and its interpretation and quantification and to describe the process in detail in the reporting of this study. The quantitative analyses only suggested configurations and guided the interpretation of the qualitative data, so the results should not be treated as ‘statistically significant’.^52^ It should be acknowledged that it cannot be interpreted from our results as to whether the use of digital health portals increased knowledge or self-care management for chronic conditions. It is also possible that factors not discussed in the interviews, such as peer-supported digital health services or specific interventions, could influence the acceptance and use of digital health services and therefore perhaps bias our clustering models.

Our sampling strategy allowed identifying this unique patient group, including users and non-users of digital health services, but the sample does not represent the entire target population. There can always be variation in qualitatively collected data, which must be considered when interpreting our results and their transferability. Special caution should be made in countries with lower levels of digitalisation and dissimilar health systems.

## Conclusion

Our study identified six clusters of acceptance and use of digital health services among persistent FAs in outpatient care that can be utilised as part of the development of guidelines for healthcare professionals to assess FAs’ suitability for digital services. Our cluster characterisation suggests that *Self-Managers* and *Telemedicine Users* with digital opportunities, awareness, and interest could most likely benefit from embedding appropriate digital services for self-management of health or telemedicine interventions into their care plan. We also identified *Supported Self-Managers* as potential users of digital services for self-management of health, as long as they are referred to secured digital support. The challenges described by *Non-Self-Managers*, *Doubtful Telemedicine Users*, and *Telemedicine Refusers* suggest that securing in-person services would be crucial for them. Even their possibilities as users of digital health services could be improved with accessible digital services and by strengthening the competence of professionals in encouraging patients to use digital services and providing high-quality services remotely.

Patient-centred promotion of the use of digital health services is challenging if the identification of the entire FA patient group is not systematic in organisations. It would be essential to identify^10^ and document discreetly those patients who continue to frequently visit outpatient care, after which different characteristics related to the acceptance and use of digital health services could be outlined. Documentation of identifiers related to frequent attendance and user patterns of digital health services should be structured so that the information is quickly visible for the following professionals to guarantee continuity of care. While digital health services are being promoted to suitable FAs, the care of those FAs who will never be able to take advantage of digitalisation must be secured by investing in in-person primary care.

## Supplemental Material

sj-docx-1-dhj-10.1177_20552076231178422 - Supplemental material for Patterns of acceptance and use of digital health services among the persistent frequent attenders of outpatient care: A qualitatively driven multimethod analysis Click here for additional data file. Supplemental material, sj-docx-1-dhj-10.1177_20552076231178422 for Patterns of acceptance and use of digital health services among the persistent frequent attenders of outpatient care: A qualitatively driven multimethod analysis by Lotta Virtanen, Anu-Marja Kaihlanen, Emma Kainiemi, Petra Saukkonen and Tarja Heponiemi in DIGITAL HEALTH

sj-docx-2-dhj-10.1177_20552076231178422 - Supplemental material for Patterns of acceptance and use of digital health services among the persistent frequent attenders of outpatient care: A qualitatively driven multimethod analysis Click here for additional data file. Supplemental material, sj-docx-2-dhj-10.1177_20552076231178422 for Patterns of acceptance and use of digital health services among the persistent frequent attenders of outpatient care: A qualitatively driven multimethod analysis by Lotta Virtanen, Anu-Marja Kaihlanen, Emma Kainiemi, Petra Saukkonen and Tarja Heponiemi in DIGITAL HEALTH
