# Supplementary material for: Patterns of acceptance and use of digital health services among the persistent frequent attenders of outpatient care: A qualitatively driven multimethod analysis
Source: Digit Health. 2023 May 25;9:20552076231178422. doi: 10.1177/20552076231178422 (PMC10226178; doi:10.1177/20552076231178422)
Supplement: sj-docx-1-dhj-10.1177_20552076231178422 - Supplemental material for Patterns of acceptance and use of digital health services among the persistent frequent attenders of outpatient care: A qualitatively driven multimethod analysis [file sj-docx-1-dhj-10.1177_20552076231178422.docx]

## Supplementary A: Interview guide

1. Warm-up: How would you describe your life situation before the corona pandemic? What health and social services did you use before the corona pandemic?

2. Have you used health services during the corona pandemic?

2.1. Have the health services you used before corona been replaced by remote services? Have you used them? Why or why not? (If no: Have you been left without health services because they have not been provided physically?)

2.2. Have most of the health services you have used during corona been provided physically or through remote connections?

3. During the corona pandemic, have you used other services, such as social services?

3.1. Have the services you used before corona been replaced by remote services? Have you used them? Why or why not? (If no: Have you been left without services because they have not been provided physically?)

3.2. Have most of the services you have used during corona been provided physically or through remote connections?

4. Could you please share your experiences of using remote health and social services during the corona pandemic?

4.1. In your opinion, have the use of remote services given you help, support or other benefits? What kind of? (If no: Why?)

4.2. How do you think remote services have functioned? What has functioned well? What has not functioned well?

4.3. How do you think remote services should be developed?

5. How has the corona pandemic affected your social relationships?

5.1. Have you experienced loneliness during the corona pandemic?

5.2. Have you used a smartphone, computer/laptop or tablet to communicate with others during corona? What device have you used? What software or platform have you used?

5.3. Do you feel that digital communication has affected your experience of loneliness? If so, which device and software/platform? How have they affected your experience of loneliness?

5.4. How could loneliness be relieved using information technology?

6. How does digitalisation influence your life now during the corona pandemic?

6.1. What thoughts and feelings have emerged for you? Could you give some examples?

7. Background questions: Age, gender, and country of birth? How many years in total have you had education?
