# Supplementary material for: Patterns of acceptance and use of digital health services among the persistent frequent attenders of outpatient care: A qualitatively driven multimethod analysis
Source: Digit Health. 2023 May 25;9:20552076231178422. doi: 10.1177/20552076231178422 (PMC10226178; doi:10.1177/20552076231178422)
Supplement: sj-docx-2-dhj-10.1177_20552076231178422 - Supplemental material for Patterns of acceptance and use of digital health services among the persistent frequent attenders of outpatient care: A qualitatively driven multimethod analysis [file sj-docx-2-dhj-10.1177_20552076231178422.docx]

## Supplementary B: Coding scheme for the data analysis

For background, participants were coded into three similar sized *age groups* (0=23–59, 1=60–69, 2=70–84 years), and by *gender* (0=men, 1=women) and *education* (0=low as maximum of a secondary education, 1=high as an academic degree). *Health status* was coded as 0=complicated if participant directly described it, for example, by mentioning a recent stroke or ongoing chemotherapy, or of receiving home care, having a disability pension, participating in rehabilitation, or living in a sheltered housing or equivalent; indicating that participant had a somatic or psychological health condition which limited the ability to function in daily life or working life. If none of these mentioned, they received 1=stable.

Table B1 shows the coding scheme for the data analysis. Establishing definitions of sub-categories helped to ensure that all the reduced expressions belonging to that sub-category had related content. The names of the sub-categories were iteratively developed and modified in phase 2, as they were used as variables in the cluster analyses.

Table B1. Coding scheme for the data analysis

| Phase 1: Qualitative analysis | | | | Phase 2: Quantification of the qualitative analysis | |
| --- | --- | --- | --- | --- | --- |
| Main category | Examples of reduced expressions | Sub-category (Variables) | Definition of sub-category | What was searched from the reduced expressions under specific sub-categories | Quantification of alternatives |
| **Use of digital health services** | ***Has not used any digital services for taking care themselves of their health matters*** | *Use of digital services for self-management of health* | Experience of digital services for self-management of health during the past 1 year (i.e., personal health tracking, seeking health information, or making transactions related to health, such as asking for a renewal of prescriptions and booking appointments) | Direct description of no experience | 0 = No |
|  | ***Has used municipal website to obtain health information*** |  |  |  |  |
|  | ***Has used digital patient platform to renew prescriptions and book appointments*** |  |  | Direct description of experience of at least one digital service for self-management of health | 1 = Yes |
|  | ***Has only received in-person health services*** | *Use of real-time telemedicine services* | Experience of real-time communication with a healthcare professional via a phone call, video call, or chat during the past 1 year | Direct description of no experience | 0 = No |
|  | ***Has asked about their symptoms and the possible need for an appointment through the municipality’s chat service*** |  |  | Direct description of experience of real-time telemedicine services at least once | 1 = Yes |
|  | ***Has received guidance on the chat of the occupational healthcare digital clinic a few times*** |  |  |  |  |
| **Facilitating conditions** | ***Has only a smart phone with a tiny screen, which makes the use as difficult*** | *End devices available* | Availability of end devices allowing the use of digital health services | Direct description of having only a smart phone which the individual perceives as difficult for using the services or describing missing sufficient end devices to use digital health services | 0 = No (Only a smart phone) |
|  | ***Due to a small pension, does not have the newest end devices, which would allow the use of digital health services*** |  |  |  |  |
|  | ***Has a laptop and a smart phone, but prefers using a laptop because of the bigger screen*** |  |  | Direct description of having at least a computer/laptop or tablet computer | 1 = Yes |
|  | ***Would like to learn to use digital services, but neither relatives nor nurses have time to help*** | *Digital support available* | Availability of user support for digital health services provided by, e.g., an organisation, a healthcare professional, or a relative | Direct description of lacking support for digital health services OR not mentioning about the support, e.g., because has advanced skills in using the services | 0 = No |
|  | ***Feels the situation as good because a skilled spouse is able to help with digital services*** |  |  | Direct description of having support for the use of digital health services or knowing where to ask help if needed | 1 = Yes |
|  | ***Knows people from whom could get support for using digital services, if wanted to use them*** |  |  |  |  |
|  | ***Feels hopelessness in the use of the Internet "although is so young"*** | *Advanced digital skills* | Ability to perform fundamental tasks on a computer or Internet | Direct description of being a beginner with information technology in general | 0 = No |
|  | ***Uses intensively different computer programmes due to his work*** |  |  | Direct description of having advanced skills to use information technology in general or perceiving the use of computers and Internet as easy, or expressions implying an intensive use of computers and Internet at work or laytime, e.g., use of websites or social media | 1 = Yes |
|  | ***Feels to be caught up in time by being able to use the patient portal, social media, and online banking themselves*** |  |  |  |  |
| **Performance expectancy** | ***Feels it is so easy to get in-person appointments that does not see the need for digital services*** | *Not needing digital health services* | Is not using digital health services because sees no need for them | Not mentioned | 0 = Not mentioned |
|  | ***Has not had any needs for using digital services, as lives close to in-person services*** |  |  | Direct description of not needing or statements, such as ‘I live close to the healthcare centre, why would I use digital health services’, ‘I am not using patient portal, as I receive all my health information on a paper’, ‘Digital health cannot provide what I need for my complex health status’, or equivalent | 1 = Yes |
|  | ***No need to use the patient portal as receives the results by post and appointments can be booked in person*** |  |  |  |  |
|  | ***Beneficial that no need to travel or wait to get an appointment at the health centre with digital services*** | *Digital health services save time and need for travelling* | Is benefitting from digital health services because their use save times and need for travelling compared to in-person services | Not mentioned | 0 = Not mentioned |
|  | ***Travelling would be a big hassle, so perceives ePrescriptions, health records, and online bookings as good services*** |  |  | Direct description or related expressions, such as ‘I am benefitting from telemedicine services because then I do not need to travel to the healthcare centre’ | 1 = Yes |
|  | ***Finds it easier and handy to use health services digitally*** |  |  |  |  |
|  | ***Likes that can be aware of what the physicians write about own health*** | *Digital health services promote the patient’s active role* | Is benefitting from digital health services because they allow to take an active role in own care | Not mentioned | 0 = Not mentioned |
|  | ***Beneficial to use patient portal to get access to own health results*** |  |  | Direct description or related expressions, such as ‘I am benefitting from patient portal because it allows me to track the results of my health examinations’ | 1 = Yes |
|  | ***Feels that it is extremely important that digital health services allow timely access to information*** |  |  |  |  |
|  | ***Perceives that phone consultations do not substitute in-person services, but provide substantial help for health*** | *Digital health services support health* | Is benefitting from digital health services because they have supported own health | Not mentioned | 0 = Not mentioned |
|  | ***Has hereditary diseases, and gets health benefits by searching related up-to-date evidence on digital platforms*** |  |  | Direct description or related expressions, such as ‘I got help for my health problem during the remote discussion with a nurse’ | 1 = Yes |
| **Effort expectancy** | ***Has not experienced problems in the use of digital services, such as the patient portal, rather finds their use as convenient*** | *Using digital health services requires high effort* | Perception or experience regarding the effort needed to use digital health services | Direct description of perceiving or experiencing that the use of digital health services is easy, or use of equivalent terms, such as convenient, fast, or handy | 0 = No |
|  | ***Finds that expressing their things remotely would be too difficult for them*** |  |  | Direct description of perceiving or experiencing that use of digital health is difficult, or use of equivalent terms, such as difficult to elaborate own expressions in telemedicine, digital health requires adopting new skills, such as to use secure e-identification and data security skills | 1 = Yes |
|  | ***Has so much going on that the need to try to learn to use digital services would be too much*** |  |  |  |  |
| **Social influence** | ***Healthcare professional referred them to use online appointment booking services*** | *Initiative to use digital health services came from others* | Important people in the social environment, such as a relative or a healthcare professional, who has encouraged to use digital health | Direct description of own initiative to find and use digital health services OR not using digital health services | 0 = No |
|  | ***A relative told about the convenience of using the patient platform to transmit own health information to healthcare*** |  |  | Direct description of having someone who has initiated/encouraged to use digital health or description that a person close to him/her is using intensively digital health services and is able to help own use too | 1 = Yes |
|  | ***Healthcare professionals have encouraged to use Health Village platform*** |  |  |  |  |
| **Hedonic motivation** | ***Not willing to familiarise themselves to use digital health services, as prioritises other things to take care in their life*** | *Positive attitude towards digital health services* | The extent of pleasure feeling from using or the thought of using digital health services | Direct description of not being interested in using digital health services or use of equivalent terms, such as not liking the use of technology or having old-fashioned preferences for the use of health services | 0 = No |
|  | ***Prefers and will use the old-fashioned health services "until the end"*** |  |  |  |  |
|  | ***Is curious about different digital services, although not yet experienced in their use*** |  |  | Direct description of having an interest in using digital health services or related expressions implying positive views towards digital health services | 1 = Yes |
